# LINC01343 targets miR-526b-5p to facilitate the development of hepatocellular carcinoma by upregulating ROBO1

**DOI:** 10.1038/s41598-023-42317-5

**Published:** 2023-10-12

**Authors:** Song Wu, Tao Tang, Hongchi Zhou, Jing Huang, Xiaoliang Kang, Junli Zhang

**Affiliations:** 1 Department of Hepatobiliary Vascular Surgery, The First Affiliated Hospital of Chengdu Medical College, Chengdu, 610500 Sichuan China; 2 Department of Pathology, The First Affiliated Hospital of Chengdu Medical College, No. 278, Baoguang Avenue, Xindu District, Chengdu, 610500 Sichuan China

**Keywords:** Cancer, Cell biology, Molecular biology

## Abstract

Long noncoding RNAs (lncRNAs) contribute to hepatocellular carcinoma (HCC) progression and development. However, the function and molecular mechanisms of action of LINC01343 in HCC remain unclear. qRT-PCR and western blotting were performed to assess miR-526b-5p, LINC01343, and ROBO1 levels in HCC cell lines and tissue samples. Flow cytometry, transwell, and cell counting kit-8 assays were conducted in vitro to assess how LINC01343 influences the apoptosis, migration, and proliferation of HCC cells. In addition, the role of LINC01343 in the growth of tumors was verified using an in vivo xenograft tumor assay. Specific binding of miR-526b-5p to LINC01343/ROBO1 was validated using RNA immunoprecipitation and dual-luciferase reporter experiments. LINC01343 was upregulated in HCC cells and tissues. In vitro, LINC01343-knockdown Hep3B and Huh-7 cells exhibited enhanced apoptosis and suppressed proliferation and migration. An in vivo study further validated that LINC01343-knockdown repressed tumor growth. In terms of mechanisms, LINC01343 directly sponged miR-526b-5p, negatively modulating its expression. Moreover, further experiments revealed that inhibiting miR-526b-5p could counteract the tumor-suppressive effects of LINC01343-knockdown in Hep3B and Huh-7 cells. ROBO1 was identified as a direct target of miR-526b-5p. ROBO1 knockdown weakens the migratory and proliferative abilities of Hep3B and Huh-7 cells. Nonetheless, the inhibition of miR-526b-5p mitigated this effect. These findings revealed that LINC01343 serves as a vital oncogene in HCC. Moreover, the LINC01343/miR-526b-5p/ROBO1 axis may be a prospective target for HCC treatment.

## Introduction

Liver cancer remains a major threat to global health^1^. It has been forecasted that, by 2025, its incidence will surpass one million cases^1,2^. Hepatocellular carcinoma (HCC) is the primary form of liver cancer^3^. Approximately 90% of liver cancer cases are attributable to HCC^3^. Although the incidence rate has declined owing to considerable advances in diagnosis, surveillance, and management, mortality rates remain high^4–6^. Transarterial chemoembolization is an effective treatment for unresectable intermediate HCC^7^, and the combination of radiofrequency ablation and transarterial chemoembolization has been reported to improve the prognosis of HCC by reducing complications^7^. However, the therapy for patients with advanced-stage HCC remains difficult. Therefore, there is an urgent need to identify efficacious biomarkers and explore new therapeutic targets and strategies for patients with HCC.

Increasing evidence indicates that HCC is a genetic and epigenetic disease^8^. Although the role of genetic alterations in HCC development has been extensively investigated, the involvement of epigenetic alterations remains relatively unexplored^9^. Long noncoding RNAs (lncRNAs) are RNA transcripts measuring over 200 nucleotides in length^10^. However, there is no evidence that these RNAs are translated into functional peptides or proteins^11,12^. Although most lncRNAs are poorly expressed, many manifest developmental stage- and tissue-specific expression patterns, indicating specific regulatory roles in various tissues^13^. LncRNAs can interact with other molecules and regulate the expression of target genes via transcriptional regulation, enhancer regulation, and sequestration of RNA or proteins^14^. Recent discoveries have proven that the participation of lncRNAs is vital for modulating multiple biological processes, including tumor development, differentiation, and metastasis^15–17^. This is possible through epigenetic mechanisms such as competing endogenous RNA (ceRNAs) that ‘sponge’ microRNAs or proteins^15–17^. Despite the discovery of thousands of lncRNAs, only a limited number have been functionally characterized^16,18^.

It has been suggested that dysregulated lncRNAs can serve as early diagnostic biomarkers for various types of cancer^19^. In HCC, lncRNAs, including CDKN2BAS, NBR2, XIST, and LINC00174, exhibit great potential for regulating metabolism, proliferation, metastasis, migration, angiogenesis, epithelial-to-mesenchymal transition (EMT), and apoptosis of cancer cells^20–23^. Nevertheless, the biological functions of most lncRNAs in HCC are yet to be explored. Identifying lncRNAs involved in driving cell transformation and promoting HCC development and progression is crucial for clarifying the mechanisms of hepatocarcinogenesis. This study provides new insights into HCC detection, diagnosis, therapeutic interventions, and prevention. Previous studies reported elevated LINC01343 levels in Ewing sarcoma and oral squamous cell carcinoma (OSCC)^24,25^. However, LINC01343’s biological functions in HCC remain unclear.

This study focused on LINC01343 and determined its function and expression patterns in HCC cells. Rescue experiments were performed to explore the molecular mechanisms underlying the LINC01343 function. LINC01343 is a promising early prognostic biomarker for HCC and provides new approaches for HCC treatment.

## Materials and methods

### Tissue samples

All the enrolled patients provided written informed consent. Patients who had undergone chemotherapy or radiotherapy before surgery were excluded from the study. Surgical procedures were performed to obtain 40 pairs of HCC tumor and adjacent non-tumor tissue specimens from patients admitted to the First Affiliated Hospital of Chengdu Medical College (Chengdu, China). All tissues were treated with liquid nitrogen and stored at − 80 °C for further studies. Two experienced pathologists validated tissue samples. The Ethics Committee of the First Affiliated Hospital of Chengdu Medical College (Chengdu, China) authorized this work. All procedures involving human participants strictly adhered to the Declaration of Helsinki and its later amendments.

### Cell culture

The HCC cell lines Huh-7, SUN-182, and Hep3B, as well as the normal hepatic cell line THLE2 were acquired from the American Type Culture Collection (ATCC, USA). All cells were grown in Dulbecco’s Modified Eagle’s Medium (DMEM; Invitrogen, USA) with 10% fetal bovine serum (FBS; Gibco, USA). The cultures were maintained at 37 °C and 5% CO_2_. When cells reached 80% confluence, they were trypsinized and passaged at 1:3.

### Small interfering RNA (siRNA) and microRNA (miRNA) inhibitor transfection

Following the manufacturer's protocol, Huh7 or Hep3B cells (5 × 10^4^ cells per well) were individually transfected with 50 nM non-targeting siRNA, si-LINC01343, si-ROBO1, miR-526b-5p inhibitor, or inhibitor control (inhibitor NC) (all synthesized by RiboBio, China) using Lipofectamine 3000 transfection reagent (Thermo Fisher, USA). Negative controls were treated with the transfection reagent alone. Forty-eight hours later, transfection efficiency was validated using qPCR. After transfection, cells were used for further experiments.

### qRT-PCR assay

TRIzol reagent (Thermo Fisher Scientific) was used to extract total cellular RNA according to the manufacturer’s protocol. Subsequently, the First Strand cDNA Synthesis Kit (Thermo Fisher Scientific) was used for the reverse transcription of the RNAs into cDNAs. The Eco Real-time PCR System (Illumina, USA) and iTaq™ Universal SYBR® Green (BioRad, USA) were used to amplify specific cDNAs. The 2^−ΔΔCT^ relative quantitative approach was applied to analyze the results, with GAPDH as the internal control.

Following the kit protocol, the miRNA Isolation Kit (OMEGA) was used to extract miRNAs. The miRcute Plus miRNA First-Strand cDNA Synthesis Kit (TIANGEN, China) was used to transcribe the miRNAs into cDNA. Using the miRcute miRNA qPCR Detection Kit (SYBR Green; TIANGEN, China), cDNAs were amplified using the Eco Real-time PCR System (Illumina). The internal control used was U6. The primer sequences are listed in Table 1.

**Table 1:** Primer sequences used in RT-qPCR.

Genes	Primer sequences
miR-526b-5p	F:5ʹ- TCGCTCTTGAGGGAAGCACT-3ʹ
	R:5ʹ- CTCAACTGGTGTCGTGGA-3ʹ
ROBO1	F:5ʹ-GCTGGCGACATGGGATCATA-3ʹ
	R: 5ʹ-AATGT GGCGGCTCTTGAACT-3ʹ
GAPDH	F: 5ʹ-AAGGTCATCCCAGAGCTGAA-3ʹ
	R: 5ʹ-CTGCTTCACCACCTTCTTGA-3ʹ
U6	F: 5ʹ-TCCCTTCGGGGACATCCG-3ʹ
	R: 5ʹ-AATTTTGGACCATTTCTCGATTTGT-3ʹ

### Subcellular fraction localization

Nuclear and cytoplasmic fractions were isolated using the PARIS Kit (Life Technologies, Inc., USA). A total of 1 × 10^7^ Huh-7 and Hep3B cells were collected, suspended, and incubated in Cell Fractionation Buffer for 10 min. After centrifugation, the pellets were resuspended in Cell Disruption Buffer. The cytoplasmic and nuclear RNAs in the buffers were extracted and analyzed using RT-qPCR to determine the LINC01343 levels. GAPDH and U6 were used as cytoplasmic and nuclear controls, respectively.

### Fluorescence in situ hybridization (FISH) assay

Fluorescence-conjugated LINC01343 probes were constructed by RiboBio (China), and the fluorescent in situ hybridization kit was purchased from RiboBio. After fixing with 4% paraformaldehyde, HCC cells were overnight hybridized with fluorescence-conjugated LINC01343 probes. The cells were stained with DAPI for 5 min in the dark. Finally, the cells were observed under a fluorescence microscope (Leica, Japan).

### Cell counting kit-8 (CCK‐8) assay

CCK-8 assay was performed to evaluate the proliferation of Hep3B and Huh-7 cells. Each well of a 96-well culture plate was inoculated with one thousand HCC cells. The cells were cultivated for 0, 24, 48, and 72 h before each well was supplied with 10 µl CCK‐8 (Dojindo, Japan). The cells were incubated for two more hours. A Multiskan Go Spectrophotometer (Thermo Fisher Scientific, USA) was used to measure the optical density (OD_450_) of the wells at a wavelength of 450 nm.

### Transwell assay

Transwell assays were performed to evaluate the migratory ability of the HCC cells. The Hep3B and Huh-7 cells were starved for 12 h. Subsequently, the top compartments of the transwell inserts were filled with 5 × 10^4^ cells and incubated in the absence of FBS. The bottom compartments were loaded with 500 μL DMEM containing 10% FBS. Following a 24-h incubation, migrated cells were fixed with formalin for 10 min before treatment with 0.1% crystal violet for 20 min at room temperature. Finally, cells were viewed and counted under a microscope (Olympus, Japan).

### Flow cytometry

Apoptosis was evaluated using flow cytometry using the FITC Annexin V Apoptosis Kit (BD Biosciences). Transfected Huh-7 and Hep3B cells (5 × 10^5^) were incubated at room temperature with 100 μL 1 × binding buffer containing 5 μL FITC Annexin V and 5 μL PI. Finally, the cells were analyzed using a flow cytometer (BD Biosciences).

### Xenograft tumor formation assay

Six-week-old male nude mice weighing 18 g were acquired from National Rodent Laboratory Animal Resources (China). Each mouse was subcutaneously injected with 5 × 10^7^ Huh-7 cells stably infected with either lentivirus sh-LINC01343 (n = 5; GeneCopoeia, USA) or sh-NC (n = 5). The volume of the tumors was determined weekly by applying the formula: volume = (width^2^ × length) ÷ 2. Five weeks after the administration of Huh-7 cells, mice were euthanized using CO_2_ before their tumors were extracted and weighed. The Ethics Committee of the First Affiliated Hospital of Chengdu Medical College (Chengdu, China) approved all the animal experiments, which were performed in accordance with the Guide for the Care and Use of Laboratory Animals.

### Dual-luciferase reporter assay

LINC01343’s target miR-526b-5p was predicted using starBase (v2.0; https://starbase.sysu.edu.cn/starbase2/index.php). Prediction of the miR-526b-5p target ROBO1 was accomplished using TargetScan (http://www.targetscan.org). Dual-luciferase reporter assays were performed to verify the binding of miR-526b-5p to LINC01343 and ROBO1. LINC01343 and ROBO1 3′UTR sequences were amplified and cloned into firefly luciferase reporter vectors (Obio Technology, China). The mutant vectors of LINC01343 or ROBO1 3′UTR were constructed by mutating the seed region of miR-526b-5p. T293 cells were transfected with a combination of 50 nM miR-526b-5p mimic, 1 μg firefly luciferase reporter vector, and 100 ng *Renilla* luciferase pRLCMV vector (Obio Technology). Forty-eight hours after transfection, a Dual-Luciferase Reporter Assay System (Promega Corporation, USA) was used to gauge the relative luciferase activity. The assay was performed according to the manufacturer’s instructions. All data from this experiment were normalized to the luciferase activity of *Renilla*.

### RNA immunoprecipitation (RIP) assay

RIP assay was performed using the Magna RIP Kit (EMD Millipore, USA). Huh-7 and Hep3B cells (1 × 10^7^) were treated with the RIP lysis buffer. The cellular extracts were exposed to magnetic beads coated overnight at 4 °C with anti-IgG antibody (EMD Millipore) or anti-Argonaute 2 antibody (AGO2, #ab32381; Abcam, USA). TRIzol (Thermo Fisher Scientific) was used for RNA extraction. The extracted RNA was analyzed using qRT-PCR.

### Western blot analysis

The Total Protein Extraction Kit (KeyGEN, China) was used to extract total proteins. The BCA kit (KeyGEN, China) was used to quantify the protein concentration. Thereafter, the proteins (30 μg) were deposited on a 10% polyacrylamide gel and transferred onto a polyvinylidene fluoride membrane. The membranes were sealed for one hour with 5% skim milk. Subsequently, the membranes were incubated overnight at 4 °C with primary antibodies against GAPDH (1:1000, ab70744, Abcam, USA) and ROBO1 (1:1000, ab245516, Abcam, USA). This was achieved by incubation at room temperature with horseradish peroxidase (HRP)-conjugated secondary antibodies (1:5000, 511,203, Zen-Bio, China). Finally, the protein signals were detected using the ECL Immobilon Western Chemiluminescent HRP Substrate (Millipore, USA) and further visualized using the ImageQuant LAS4000 mini (GE Healthcare, USA).

### Statistical analysis

All statistical tests were performed using GraphPad Prism 8. Results were presented as mean ± SD. Student’s t-test and one-way ANOVA plus Sidak’s or Tukey’s test were used to test the significance of variations between and among groups, respectively. Linear regression analysis was performed to analyze the relationship between miR-526b-5p, ROBO1, and LINC01343. Statistical significance was set at *P* < 0.05.

### Ethics approval

The Ethics Committee of the First Affiliated Hospital of Chengdu Medical College (Chengdu, China) granted approval to this work [grant number: 2021CYFYIRB-HA-82]. The clinical tissue specimen processing complied with the ethical standards of the Declaration of Helsinki. A written informed consent was signed by each patient. The Animal Care and Use Committee of the First Affiliated Hospital of Chengdu Medical College authorized the animal experiments. The animal studies accomplished in this work were executed in strict adherence to the ARRIVE guidelines.

### Consent to participate

Each patient signed a written informed consent. Copies of their written consent are available for review by the Editor-in-Chief of this journal upon request.

## Results

### LINC01343 is highly expressed in HCC cells and tissues

**Figure 1:**
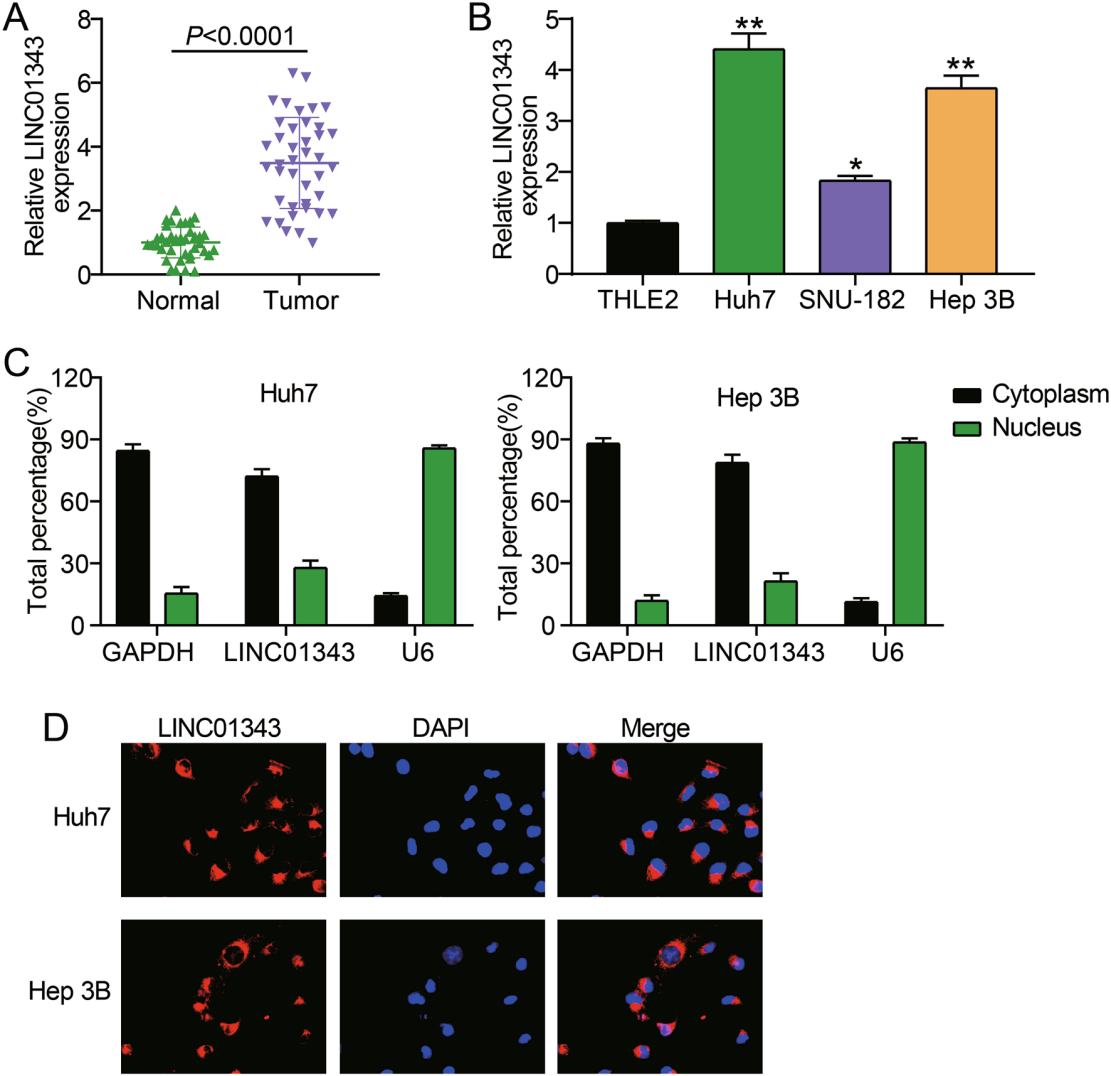
LINC01343 was upregulated in HCC. (**A**) qRT-PCR analysis of LINC01343 expression in 40 pairs of HCC tumor tissues and the adjacent normal tissues. (**B**) qRT-PCR analysis of LINC01343 expressions in HCC cells (Huh‐7, Hep3B and SNU-182) and normal liver epithelial THLE2 cells *P < 0.05 and **P < 0.001 versus THLE2 cells group. (**C**) LINC01343 expression in subcellular fractions of Huh-7 and Hep3B cells detected by real-time PCR, with U6 and GAPDH as nuclear and cytoplasmic markers, respectively. (**D**) LINC01343 expression in subcellular fractions of Huh-7 and Hep3B cells detected by FISH assay.

LINC01343 levels in HCC were determined using qRT-PCR. LINC01343 expression was 3.6-fold higher in HCC tissues than in the adjacent non-tumor tissues (Fig. 1A). The expression of LINC01343 in HCC cell lines (Hep3B, Huh‐7, and SNU-182) and normal liver epithelial cells (THLE2) was analyzed. We observed the upregulation of LINC01343 in HCC cell lines. Huh‐7 and Hep3B cells manifested the highest expression of LINC01343, hence they were chosen for the succeeding experiments (Fig. 1B). Considering that cytoplasmic and nuclear lncRNAs have different functions^13^, we performed subcellular fractionation and FISH assays to determine the localization of LINC01343 in Hep3B and Huh-7 cells. The subcellular fractionation assay revealed that approximately 70% of LINC01343 was enriched in the cytoplasm (Fig. 1C). The results of the FISH assay further demonstrated that LINC01343 predominantly existed within the cytoplasm (Fig. 1D). Our results suggested that LINC01343 is localized in the cytoplasm and is likely to be involved in the epigenetic regulation of protein translation or transport.

### Silencing LINC01343 diminished the capacities of the HCC cells to migrate and proliferate, stimulated their apoptosis, and suppressed tumor growth in vivo

**Figure 2:**
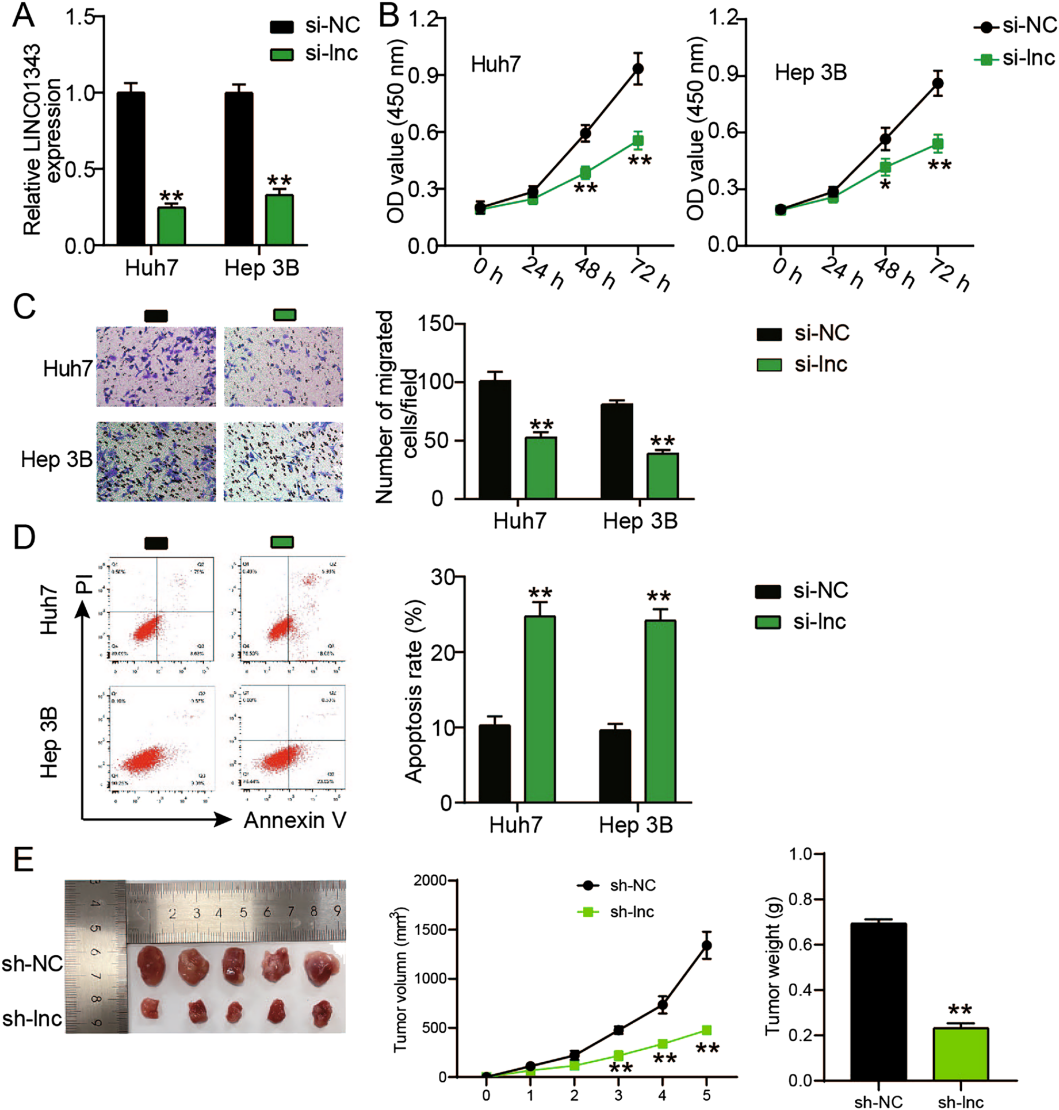
LINC01343 downregulation inhibited HCC progression both in vitro and in vivo. (**A**) qRT-PCR analysis of LINC01343 expression in Huh-7 and Hep3B cells after si-LINC01343 or si-NC transfection (n = 3). (**B**) CCK-8 experiment was performed to evaluate the proliferative ability of Huh-7 and Hep3B cells that had been transfected with si-LINC01343 or si-NC (n = 3). (**C**) Transwell experiments were conducted to assess the invasive ability of Huh-7 and Hep3B cells that had been transfected with si-LINC01343 or si-NC (n = 3). (**D**) Cell apoptosis of Huh-7 and Hep3B cells transfected with si-NC or si-LINC01343 were examined by Annexin V-FITC/PI-labeled flow cytometry (n = 3). (**E**) Animal study was performed to determine the role of LINC01343 knockdown in vivo (n = 5/group). *P < 0.05 and **P < 0.001 versus si-NC or sh-NC group.

To clarify the function of LINC01343 in HCC, it was silenced in Huh‐7 and Hep3B cells (Fig. 2A). We observed a decrease in LINC01343 expression in Huh‐7 and Hep3B cells transfected with si-LINC01343. In vitro assays were conducted to determine whether LINC01343 directly influenced the malignant phenotypes of HCC cells. The CCK-8 assay uncovered that silencing LINC01343 in Hep3B and Huh‐7 cells remarkably diminished their proliferative capacity (Fig. 2B). Moreover, transwell assays demonstrated that LINC01343 inhibition decreased the number of migratory Hep3B and Huh‐7 cells by approximately 50%, indicating that LINC01343 inhibition impeded cell migration (Fig. 2C). Using flow cytometry, we observed that LINC01343-knockdown elevated the apoptosis rate 2.5-fold in Hep3B and Huh‐7 cells (Fig. 2D). To assess the potential effects of LINC01343 on HCC tumor growth in vivo, Huh-7 cells stably transfected with sh‐LINC01343 were injected into nude mice. The sizes of the xenografts were calculated weekly. The results show that the knockdown of LINC01343 suppressed tumor volume. The weights of the xenografts were recorded. The weights of tumors from the sh-LINC01343 group were reduced by more than 60% compared with those in the sh-NC group (Fig. 2E). Our findings indicated that LINC01343 serves as a pro-oncogenic regulator of HCC progression.

### LINC01343 sponged miR-526b-5p

**Figure 3:**
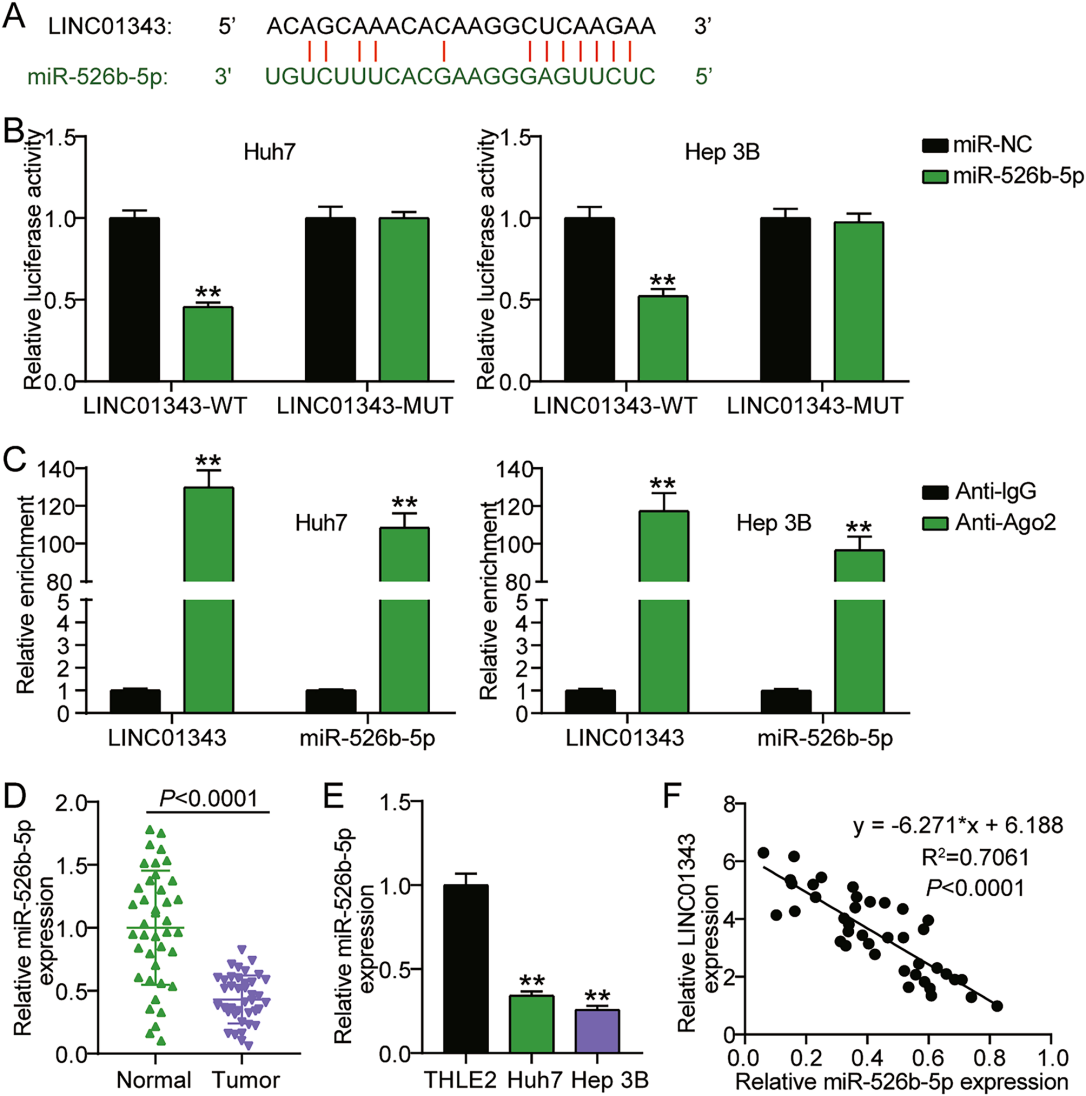
MiR-526b-5p was a target of LINC01343. (**A**) The miR-526b-5p and LINC01343 binding sites were identified using StarBase (https://starbase.sysu.edu.cn/starbase2/index.php). (**B**) The relationship between miR-526b-5p and LINC01343 was confirmed by performing dual-luciferase reporter assay among the Huh-7 and Hep3B cells (n = 3). **P < 0.001 versus miR-NC group. (**C**) The relationship between miR-526b-5p and LINC01343 was confirmed by carrying out RIP experiments among the Huh-7 and Hep3B cells (n = 3). **P < 0.001 versus Anti-IgG group. (**D**) qRT-PCR analysis of miR-526b-5p expressions in 40 sets of HCC tumor samples and the adjacent non-tumor tissues. (**E**) qRT-PCR analysis of miR-526b-5p expressions in HCC cells (Hep3B and Huh-7) and normal THLE2 cells (n = 3). **P < 0.001 versus THLE2 cells group. (**F**) Linear regression analysis of the association between LINC01343 and miR-526b-5p expressions in HCC tissues.

As LINC01343 is predominantly located in the cytoplasm of HCC cells, it may operate as a ceRNA to bind certain miRNAs and improve target gene expression. The bioinformatics tool starBase was used to predict target miRNAs putatively bound to LINC01343. Based on these results, miR-526b-5p may be a potential LINC01343 target. LINC01343/miR-526b-5p binding sites are shown in Fig. 3A. Luciferase activity experiments were performed to validate the binding sites. We observed that the Huh‐7 and Hep3B cells exhibited 50% luciferase reporter activities following their transfection with a combination of a miR-526b-5p mimic and wild-type (WT) LINC01343. By contrast, co-transfection of the LINC01343 mutant (MUT) and miR-526b-5p mimic into Huh‐7 and Hep3B cells barely affected luciferase activity (Fig. 3B). These data suggested that LINC01343 and miR-526b-5p bind together. To further validate their direct binding, we performed RIP assays on Huh‐7 and Hep3B cells. The transfection of the miR-526b-5p mimic increased LINC01343 levels by approximately 120-fold in RNAs enriched with AGO2. This suggested that miR-526b-5p and LINC01343 interact with each other (Fig. 3C). We examined miR-526b-5p expression in HCC cell lines and tissues. The results suggested that, unlike the control groups, the HCC tissues (Fig. 3D) and cell lines (Fig. 3E) showed downregulation of miR-526b-5p by more than 50%. miR-526b-5p expression was negatively correlated with LINC01343 expression (Fig. 3F). Taken together, these findings indicated that LINC01343 may function as a ceRNA to modulate miR-526b-5p expression.

### miR-526b-5p mediated the oncogenic role of LINC01343 on HCC cells

**Figure 4:**
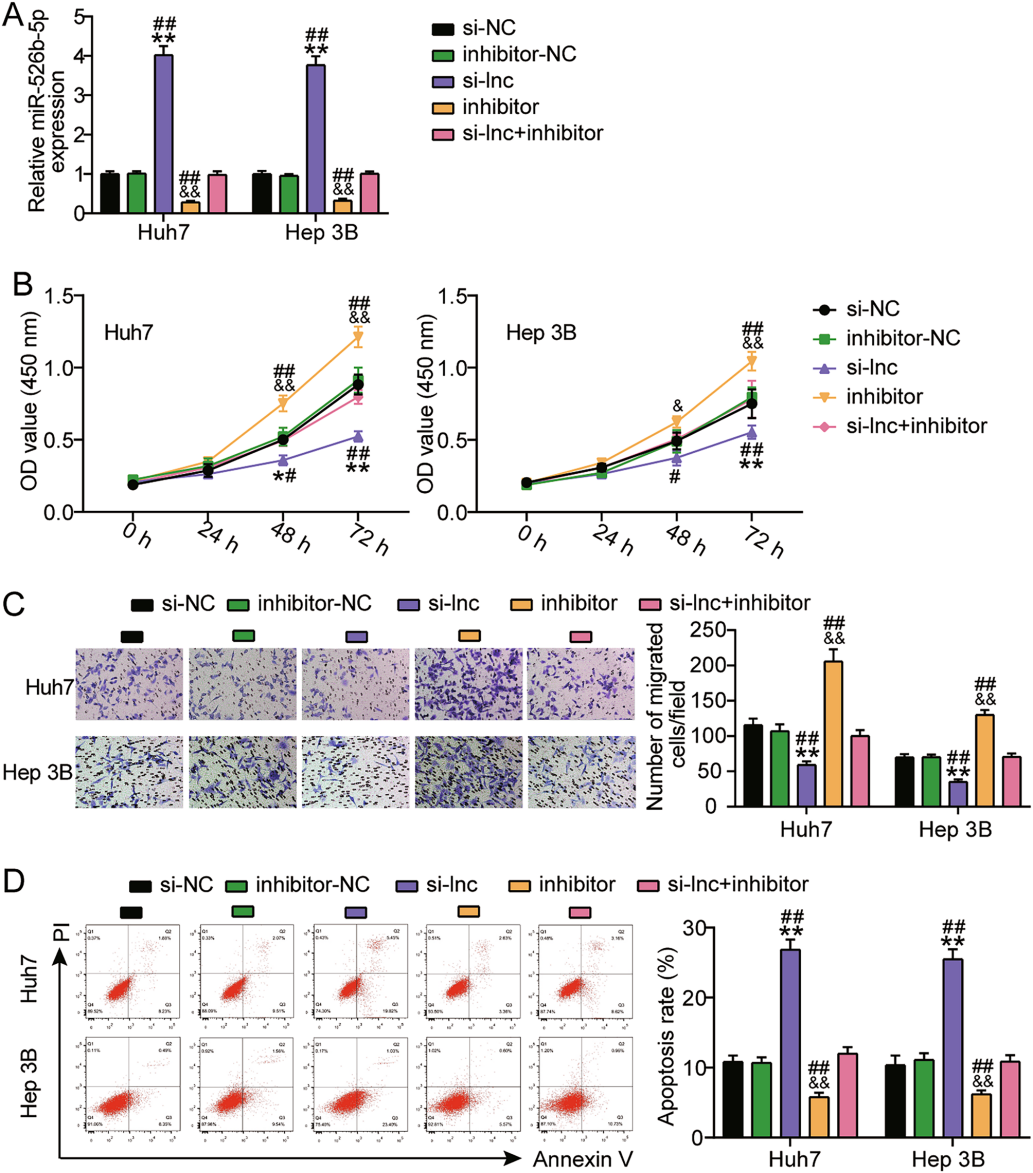
MiR-526b-5p moderated LINC01343’s oncogenic role in HCC cells. (**A**) Relative miR-526b-5p expressions in Hep3B and Huh-7 cells after si-LINC01343/siRNA control (si-NC) and/or miR-526b-5p inhibitor/inhibitor control (inhibitor-NC) transfection, as analyzed via qRT-PCR. (**B**) CCK-8 experiment was performed to evaluate the proliferative ability of Huh-7 and Hep3B cells that had been transfected with or without si-LINC01343 and/or miR-526b-5p inhibitor. (**C**) Transwell experiments were performed to assess the migratory ability of Hep3B and Huh-7 that did or did not have si-LINC01343 and/or miR-526b-5p inhibitor transfection. (**D**) Apoptosis of Huh-7 and Hep3B cells that had been transfected with or without si-LINC01343 and/or miR-526b-5p inhibitor were evaluated through Annexin V-FITC/PI-labeled flow cytometry. *P < 0.05 and **P < 0.001 versus the si-NC group; ^##^P < 0.05 and ^##^P < 0.001 versus the inhibitor-NC group; ^&^P < 0.05 and ^&&^P < 0.001 versus the si-lnc + inhibitor group.

The relationship between LINC01343 and miR-526b-5p expression was further investigated. First, either miR-526b-5p or LINC01343 was silenced in the HCC cell lines. The knockdown of LINC01343 significantly upregulated miR-526b-5p by approximately fourfold, but this outcome was offset by introducing the miR-526b-5p inhibitor (Fig. 4A). These findings indicated that miR-526b-5p and LINC01343 were successfully inhibited in Hep3B and Huh‐7 cells. Functional analysis revealed that miR-526b-5p inhibitor robustly enhanced Hep3B and Huh‐7 cell proliferation. Furthermore, silencing LINC01343 resulted in a decline in proliferation, which was reversed by inhibiting miR-526b-5p (Fig. 4B). Similarly, inhibition of miR-526b-5p also restored the LINC01343-knockdown-repressed migration of Hep3B and Huh‐7 cells (Fig. 4C). Additionally, following miR-526b-5p silencing, apoptosis was decreased in Hep3B and Huh-7 cells with LINC01343 knockdown (Fig. 4D). Overall, miR-526b-5p mediated LINC01343’s oncogenic function during HCC progression.

### ROBO1 was a miR-526b-5p target gene

**Figure 5:**
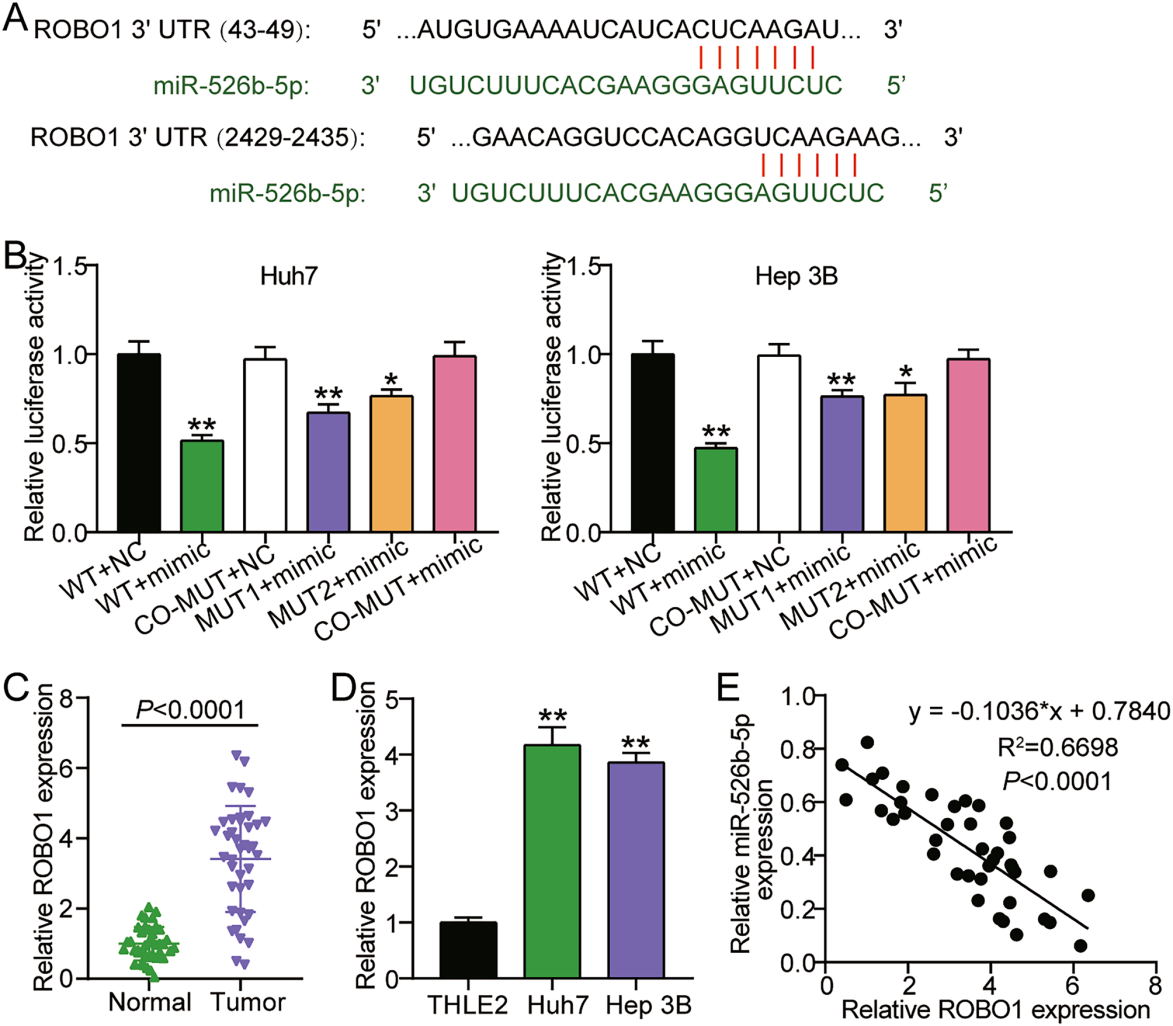
ROBO1 was a target of miR-526b-5p. (**A**) The binding sites between miR-526b-5p and ROBO1 were predicted by TargetScan. (**B**) The relationship between miR-526b-5p and ROBO1 was confirmed by dual-luciferase reporter assay in Hep3B and Huh-7 cells (n = 3). *P < 0.05 and **P < 0.001 versus wt group. (**C**) qRT-PCR analysis of ROBO1 expression in 40 pairs of HCC tumor tissues and the adjacent normal tissues. (**D**). qRT-PCR analysis of ROBO1 expressions in the HCC cells (Hep3B and Huh-7) and normal THLE2 cells (n = 3). **P < 0.001 versus THLE2 cells group. (**E**) Linear regression analysis of the association between LINC01343 and miR-526b-5p expressions in HCC tissues.

To further probe the molecular mechanisms underlying the biological function of miR-526b-5p in HCC, TargetScan was used to predict target genes. ROBO1 and miR-526b-5p had two predicted binding sites (Fig. 5A). miR-526b-5p’s binding sites on ROBO1 were further confirmed using luciferase reporter assays in Hep3B and Huh‐7 cells (Fig. 5B). The results revealed that miR-526b-5p mimics considerably repressed luciferase activity by 50% in cells that had been transfected with the WT ROBO1 vector. Nevertheless, these effects were abolished by the mutant variants. These results indicate that miR-526b-5p could directly target the ROBO1 3′UTR. We assessed ROBO1 expression in HCC cell lines and tissues. ROBO1 expression was upregulated 3.4-fold in HCC tissues (Fig. 5C) and approximately fourfold in HCC cell lines (Fig. 5D). ROBO1 expression negatively correlated with miR-526b-5p expression (Fig. 5E). These results suggested that ROBO1 serves as a miR-526b-5p target.

### miR-526b-5p targeted ROBO1 to suppress HCC cell migration and proliferation and stimulate apoptosis

**Figure 6:**
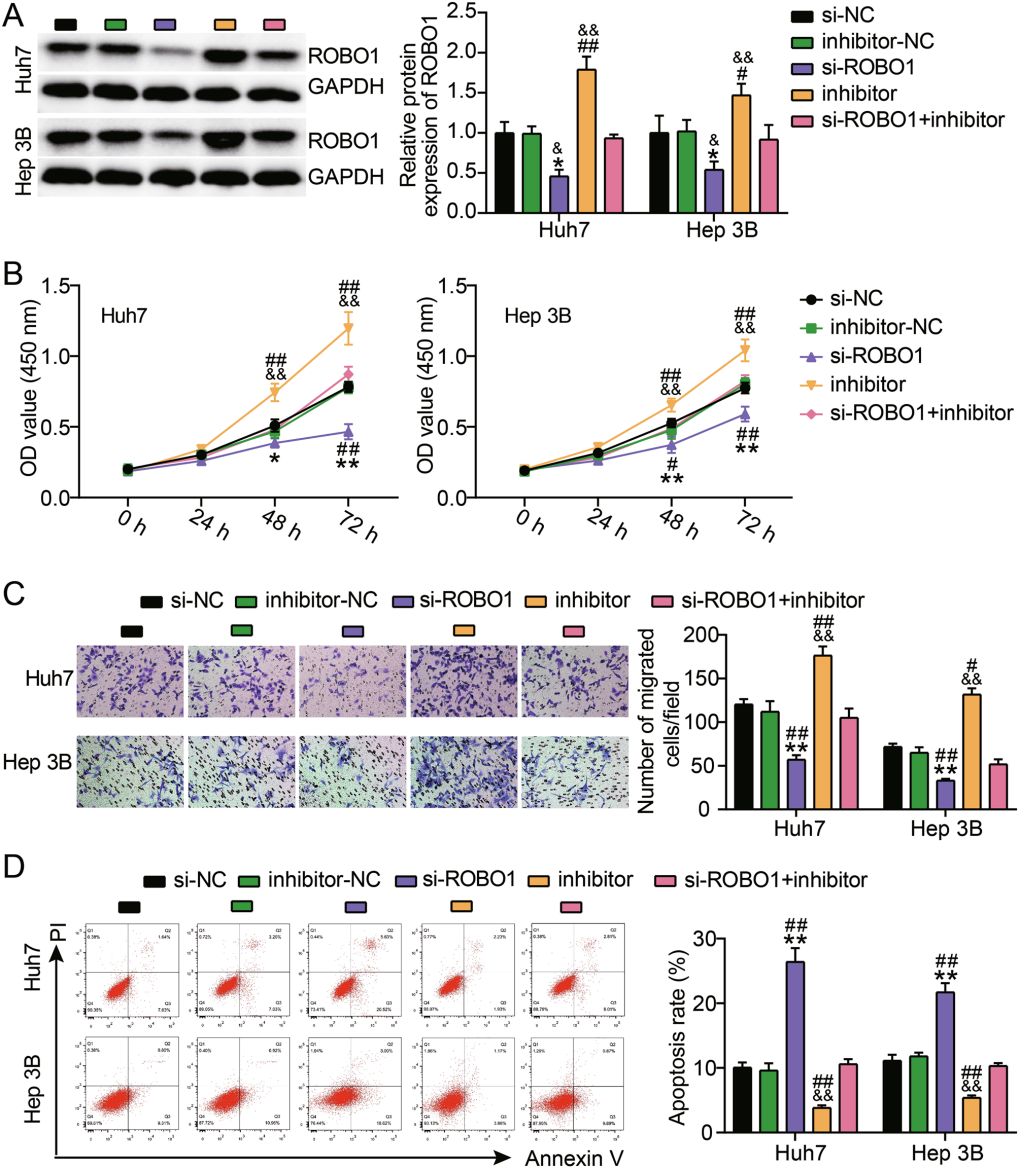
MiR-526b-5p inhibited proliferation and migration while promoted apoptosis of HCC via targeting ROBO1. (**A**) Western blot analysis of ROBO1 expressions in Huh-7 and Hep3B cells after si-DLGAP5/siRNA (si-NC) and/or miR-526b-5p inhibitor/inhibitor control (inhibitor-NC) transfection. (**B**) CCK-8 experiment was performed to evaluate the proliferative ability of Huh-7 and Hep3B cells that had been transfected with or without si-ROBO1 and/or miR-526b-5p inhibitor. (**C**) Transwell experiments were conducted to assess the migratory abilities of Hep3B and Huh-7 cells that did or did not have si-ROBO1 and/or miR-526b-5p inhibitor transfection. (**D**) Apoptosis of Hep3B and Huh-7 cells that had or had not been transfected with si-ROBO1 and/or miR-526b-5p inhibitor were assessed through Annexin V-FITC/PI-labeled flow cytometry. *P < 0.05 and **P < 0.001 versus the si-NC group; ^#^P < 0.05 and ^##^P < 0.001 versus the inhibitor-NC group; ^&&^P < 0.001 versus the si-ROBO1 + inhibitor group.

The interaction between ROBO1 and miR-526b-5p during HCC progression was further explored. First, either miR-526b-5p or ROBO1 was silenced in the HCC cell lines. Western blot analysis demonstrated that miR-526b-5p knockdown remarkably upregulated ROBO1 expression 1.97-fold in Hep3B cells and 1.68-fold in Huh‐7 cells. Furthermore, ROBO1 silencing alleviated the upregulation caused by the miR-526b-5p knockdown (Fig. 6A). Although the knockdown of ROBO1 in HCC cells suppressed proliferation, this effect was offset when the miR-526b-5p expression was inhibited (Fig. 6B). Similarly, miR-526b-5p inhibition restored migration induced by ROBO1 knockdown in Hep3B and Huh‐7 cells (Fig. 6C). Additionally, when miR-526b-5p was silenced, apoptosis declined in the Hep3B and Huh-7 cells that had ROBO1 knockdown (Fig. 6D). These findings indicated that by targeting ROBO1, miR-526b-5p represses the migration and proliferation of HCC cells while promoting their apoptosis.

## Discussion

HCC is one of the most common cancer types worldwide and is currently the third leading cause of cancer-associated deaths^26,27^. Despite immense efforts to reduce mortality, the exact mechanisms underlying HCC initiation and progression remain unclear^28^. This study documented the upregulation of LINC01343 in HCC cells and tissues. LINC01343 knockdown repressed the migration and proliferation of HCC cells while boosting their apoptosis. With regard to its mechanism, LINC01343 sponged miR-526b-5p to upregulate ROBO1. Overall, LINC01343 has the potential to serve as a novel biomarker for HCC prognosis and treatment.

Although most lncRNAs have poorer expression levels than protein-coding genes, many are tissue-specific and dysregulated under various pathological conditions^17^. Various lncRNAs have been shown to have aberrant expression levels and contribute to HCC tumorigenesis and progression^29^. Yang et al. have reported that 107 metastasis-associated lncRNAs are deregulated in HCC^30^. Previous studies have reported that LINC01343 expression is altered in Ewing’s sarcoma and oral squamous cell carcinoma. Nonetheless, the biological functions of LINC01343 remain unclear^24,25^. There have been no systematic analyses of the role of LINC01343 in tumors such as HCC. Further validation studies are required to confirm this hypothesis. In this study, we report that LINC01343 is a novel lncRNA that regulates cell proliferation, reduces apoptosis, and contributes to HCC progression. Many studies have shown that changes in lncRNA expression promote cancer progression by accelerating cancer cell proliferation, increasing resistance to cell death, and promoting angiogenesis^31^. Multiple lncRNAs have been shown to promote HCC cell proliferation and contribute to HCC growth through various mechanisms. For example, adipose triglyceride lipase is expressed in human HCC tissues and cells^32^. Through adipose triglyceride lipase and its products, FFA, and DAG, lncRNA-NEAT1 can promote HCC cell growth by disrupting lipolysis in HCC cells^32^. FAM83H-AS1 is upregulated in HCC tissues^33^. Knocking it down significantly repressed the proliferation of HCC cells by modulating the Wnt/β-catenin pathway^33^. lncRNAs can also serve as regulators of apoptosis and contribute to the progression of HCC. For example, lncRNA-PDPK2P has been observed to interact with PDK1 and promote the progression of HCC via the PDK1/AKT/caspase 3 signaling pathway^34^. Herein, we also found that LINC01343 could reduce apoptosis in HCC cells. The outcomes of this study suggest that LINC01343 may be a useful clinical biomarker and therapeutic target for the diagnosis, prognosis, and treatment of HCC.

lncRNAs are thought to modulate the expression of genes by interacting with miRNAs as “sponges” and attenuating the repressive effect of miRNAs on mRNAs^35^. Through this crosstalk, lncRNAs and miRNAs form a complex and control apoptosis, metabolism, proliferation, metastasis, and drug resistance of HCC cells^36^. Herein, we demonstrated that LINC01343 sponges miR-526b-5p in HCC cells. Previous studies have documented that miR-526b-5p functions as a tumor suppressor in the formation and development of a variety of cancers, such as oral squamous cell carcinoma, esophageal squamous cell carcinoma, osteosarcoma, melanoma, lung cancer, and gastric cancer^37–40^. For instance, in colon cancer, miR-526b-5p downregulates HIF-1α, impedes glycolysis, and represses cancer cell metastasis and proliferation^41^. Another study has shown that miR-526b targets WEE1 and suppresses glioma cells^42^. Moreover, previous studies have reported that miR-526b-5p operates via a ceRNA network with tumor-related noncoding RNAs, including circular RNAs (circ_UHRF1, circ_SPECC1, circ_0085539, circ_UGGT2, and circ_0091581) and lncRNAs (NCK1-AS1)^37,39,43–45^. For example, in melanoma, miR-526b-5p binds to NCK1-AS1 to facilitate tumor cell migration and proliferation^37^. However, this effect was reversible through miR-526b-5p depletion^37^. miR-526b-5p downregulation in HCC suppresses cell proliferation and migration^44^. Mechanistically, miR-526b-5p binds to circ_UGGT2, and the inhibitory effect of circ_UGGT2 silencing on HCC progression is impaired by overexpressing miR-526b-5p^44^. In our study, we also observed miR-526b-5p downregulation in HCC cells. Furthermore, miR-526b-5p knockdown reversed the inhibitory effects of LINC01343 knockdown on HCC cell growth. Our findings suggested that LINC01343 influences HCC progression by targeting miR-526b-5p.

We demonstrated that ROBO1 was a target of miR-526b-5p. ROBO1 is a conserved transmembrane receptor protein primarily found in the nervous system^46^. It is also expressed in other tissues and cells, such as muscle and vascular endothelial cells^46^. ROBO1 is a receptor for SLIT1 and SLIT2, which mediate cellular responses and provide molecular guidance cues during cell migration^47^. Previous studies have reported that ROBO1 promotes the development of HCC via various mechanisms. SLIT2 knockdown upregulates the expression of ROBO1 and then partially promotes HCC invasion by upregulating MMP2 through activation of the PI3K pathway^48^. In colorectal epithelial cell carcinogenesis, the SLIT2/ROBO1 signal can also recruit Hakai, a ubiquitin ligase, to induce the ubiquitination and lysosomal degradation of E-cadherin, stimulating EMT^49^. Moreover, ROBO1 promotes angiogenesis in HCC and proliferation, motility, and tube formation in HUVECs. ROBO1 increases the expression of cell division cycle 42 and distorts the actin cytoskeleton in HUVECs^50^. These results suggest that ROBO1 promotes HCC development through various mechanisms, such as stimulation of HCC cell migration, proliferation, and invasion, as well as stimulation of angiogenesis. Our findings are consistent with the ROBO1 results mentioned earlier. This study demonstrated that ROBO1 knockdown strongly inhibited HCC cell proliferation and migration while boosting apoptosis. Furthermore, miR-526b-5p inhibited HCC progression by reducing the expression of ROBO1.

Our study revealed elevated LINC01343 levels in HCC. Moreover, the knockdown of LINC01343 in HCC cells reduced cell migration and proliferation while stimulating apoptosis. LINC01343 targeted miR-526b-5p to upregulate ROBO1 expression. These findings revealed that LINC01343 serves as a vital oncogene in HCC. Furthermore, the LINC01343/miR-526b-5p/ROBO1 axis holds promise as a future therapeutic target for HCC (Supplementary Information S1).

### Supplementary Information

Supplementary Information.

## Data Availability

All data that have been created or analyzed in the course of this study have been appended to this article.
