# Supplementary figures and images for: LINC01343 targets miR-526b-5p to facilitate the development of hepatocellular carcinoma by upregulating ROBO1
Source: Sci Rep. 2023 Oct 12;13:17282. doi: 10.1038/s41598-023-42317-5 (PMC10570363; doi:10.1038/s41598-023-42317-5)

**Huh7 ROBO1 for Fig 6A**

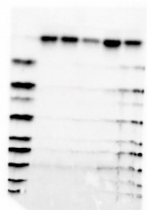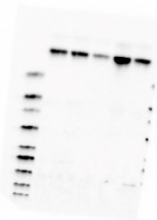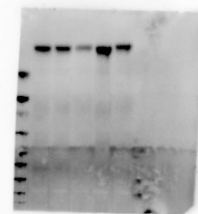

**Huh7 GAPDH for Fig 6A**

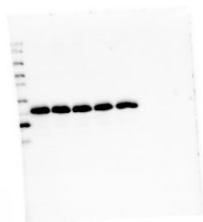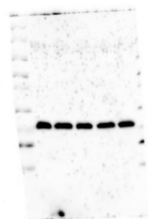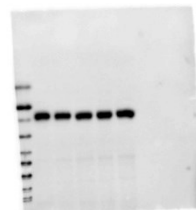

**Hep3B ROBO1 for Fig 6A**

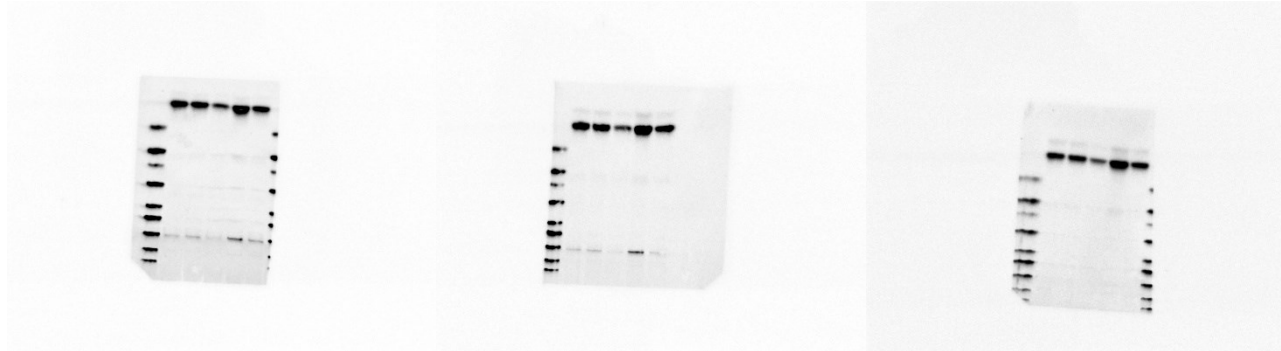

**Hep3B GAPDH for Fig 6A**

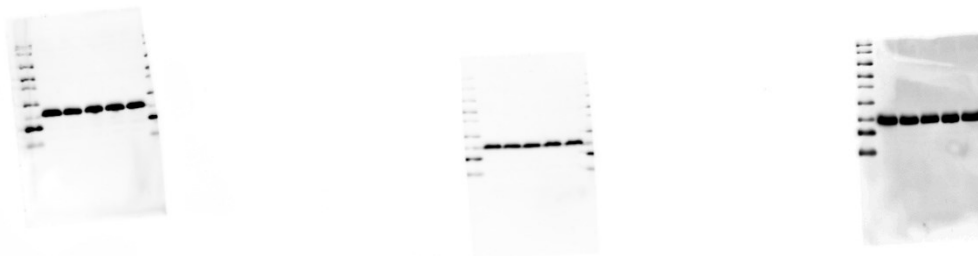

Supplement: Supplementary file 1 — Supplementary Information. [file 41598_2023_42317_MOESM1_ESM.pdf]
